# Targeted Metabolomic Analysis of Polyphenols with Antioxidant Activity in Sour Guava (*Psidium friedrichsthalianum* Nied.) Fruit

**DOI:** 10.3390/molecules22010011

**Published:** 2016-12-23

**Authors:** Carmen Tatiana Cuadrado-Silva, Maria Ángeles Pozo-Bayón, Coralia Osorio

**Affiliations:** 1 Departamento de Química, Universidad Nacional de Colombia, AA 14490 Bogotá, Colombia; ctcuadrados@unal.edu.co; 2 Instituto de Investigación en Ciencias de la Alimentación (CIAL) (CSIC-UAM), C/Nicolás Cabrera, 9, Campus de Cantoblanco, 28049 Madrid, Spain; m.delpozo@csic.es

**Keywords:** myrtaceae, tropical fruit, metabolomics, targeted analysis, ellagic acid

## Abstract

*Psidium* is a genus of tropical bushes belonging to the Myrtaceae family distributed in Central and South America. The polar extract of *Psidium friedrichsthalianum* Nied. was partitioned with ethyl ether, ethyl acetate, and *n*-butanol, and the total phenolic content and antioxidant activity were measured by Folin-Ciocalteu and ABTS assays, respectively. The ethyl acetate fraction exhibited both the highest phenolic content and antioxidant activity. Due to the complexity of this fraction, an analytical method for the comprehensive profiling of phenolic compounds was done by UPLC-ESI/QqQ in MRM (multiple reaction monitoring) mode. In this targeted analysis, 22 phenolic compounds were identified, among which several hydroxybenzoic, phenylacetic, and hydroxycinnamic acid derivatives were found. This is the first time that (+)-catechin, procyanidin B1, procyanidin B2, and (−)-epicatechin have been reported as constituents of sour guava. A fractionation by exclusion size, C_18_-column chromatography, and preparative RRLC (rapid resolution liquid chromatography) allowed us to confirm the presence of ellagic acid and isomeric procyanidins B, well-known bioactive compounds. The content of phenolic compounds in this fruit shows its potential for the development of functional foods.

## 1. Introduction

The genus *Psidium*, belonging to the Myrtaceae family, is native to the Caribbean. The specie most known, cultivated, and consumed worldwide is the common guava (*Psidium guajava* L.). This fruit is often included within the category of “superfruits”, because it exhibits antidiarrheal, antimicrobial, antioxidant, and hepatoprotective activities, among others. It has a high content of bioactive compounds [1], among which phenolic compounds are relevant [2,3]. There are other species commonly named Cas guava or sour guava (*Psidium friedrichsthalianum* Nied.), whose fruits are attractive for their organoleptic properties [4], and they have not been extensively studied. The origin of this species is Central America, but nowadays it is cultivated in different tropical countries, such as Colombia, Brazil, and Ecuador. Its non-technical cultivation in Colombia has spread from north to central regions, where it is mainly used to prepare juices. Flores et al. [5] reported the presence of phenolic compounds (flavonoids and ellagic acid derivatives) in this fruit exhibiting antioxidant properties. The phenolic-rich fraction showed inhibitory activity on IL-8 (interleukin-8) production and MMP-1 (matrix metalloproteinase-1) expression, with potent activity in both assays (100 µg/mL).

It is well known that most free radicals and reactive oxygen species (ROS: chemically reactive oxygen-containing molecules) are considered harmful because they are involved in the development of different diseases, such as cancer, inflammatory processes, and Alzheimer´s disease, among others [6,7]. Some phenolic compounds have antioxidant activity due to their ability as free radical scavengers, such as hydrogen donors, and their potential as reducing agents and/or chelating agents.

Phenolic compounds are one of the most numerous and representative groups of bioactive compounds present in most fruits and vegetables used for human consumption [8]. They have been characterized using common spectroscopic techniques such as UV-Vis, IR, mass spectrometry (MS) and NMR. When present in mixtures, they also have been analyzed by using hyphenated chromatographic techniques such as HPLC-UV-Vis, HPLC-ELSD (evaporative light scattering detector), and HPLC-MS. In this regard, the metabolomic approach has been very helpful because it was designed to identify and quantify all metabolites in a sample, in less time than the usual way and without purification, thus contributing to the understanding of complex molecular interactions in biological systems, achieving an overview within a shorter time [9,10,11]. In addition, targeted metabolomics represents an attractive strategy for food analysis. This methodology aims to quantify a predefined set of metabolites, typically dozens or hundreds of known compounds, based on metabolite-specific signals. In particular, in targeted metabolomics approaches, using triple-quadrupole mass spectrometers, a precursor ion and a product of the precursor ion, producing a molecular weight and structure-specific measurement for a single metabolite (referred to as transition), are used for the sensitive and accurate determination of the compound concentration over a wide dynamic range. Simultaneous analysis of multiple transitions results in multiple reaction monitoring (MRM).

This strategy has been successfully applied for the quantification of multiple classes of phenolic compounds in different types of fruits and beverages [12]. Thus, the main aim of this work was to identify the phenolic chemotype compounds present in *Psidium friedrichsthalianum* fruits by using traditional spectroscopic analyses as well as a targeted metabolomic approach, as they can act as antioxidants in food products developed from this fruit.

## 2. Results and Discussion

### 2.1. Fractionation of Sour Guava Extract

The ripeness parameters of the fruits used were: pH 2.67 ± 0.08, soluble solid content 9.6 ± 0.3 °Brix, and titratable acidity 2.75% ± 0.08% of citric acid. This sour guava is more acidic than the common guava (*Psidium guajava* L.). To choose the more efficient separation method, preliminary tests with different solid stationary phases (RP-18, Amberlite XAD-4, and Toyopearl HW-40S) and solvents were performed; however, the procedure published by Isaza et al. [13], the successive extraction with solvents of increasing polarity, allowed us to get four fractions with a good percentage recovery. The major fraction was the aqueous, corresponding to 11.7% by weight of the fresh whole fruits. These fractions were submitted to Folin-Ciocalteu and ABTS assays in order to select those fractions exhibiting the highest values (Table 1). These results showed that the fraction with the highest content of phenolic compounds was the F.EtOAc fraction, and it was also the fraction exhibiting the highest antioxidant activity under the ABTS assay. This result is in agreement with that reported by Flores et al. [5], despite the extraction procedure being different. In contrast, the aqueous fraction also showed a large amount of phenolic compounds, but a small value of scavenger activity. This fraction could contain a large amount of sugars and organic acids, which can interfere in the determination of phenolic compounds by the Folin-Ciocalteou method.

Thus, EtOAc was selected for further LC-MS analyses and fractionation. The fractionation by the exclusion size mechanism (Toyopearl HW-40S) allowed us to get four subfractions (F.EtOAc.1–F.EtOAc.4), among which F.EtOAc.1 and F.EtOAc.2 were shown to have the highest content of phenolic compounds. HPLC-MS analyses of the four fractions revealed that F.EtOAc.3 and F.EtOAc.4 were very complex mixtures with the presence of polymeric compounds with a MW from 600 to 1000 u. For that reason, a targeted metabolomics analysis on the F.EtOAc fraction was performed to identify some of the phenolic compounds in this fraction.

### 2.2. Targeted Analysis of Phenolic Compounds in Ethyl Acetate Fraction

Targeted HPLC-MS analyses were performed taking into account a variety of phenolic compounds, such as benzoic acid derivatives, flavan-3-ols, phenylpropanoic acid derivatives, phenylacetic acid derivatives, valeric acid derivatives, and other aromatic compounds. With UPLC-ESI/QqQ analysis, we identified and quantitated 22 phenolic compound types (Table 2).

The major ones were: (+)-catechin, procyanidin B1, procyanidin B2, (−)-epicatechin, and ellagic acid. All of these compounds, except ellagic acid, are reported for the first time in this work as constituents of sour guava (*Psidium friedrichsthalianum* Nied.).

### 2.3. Fractionation of Ethyl Acetate Fraction

**Figure 1:**
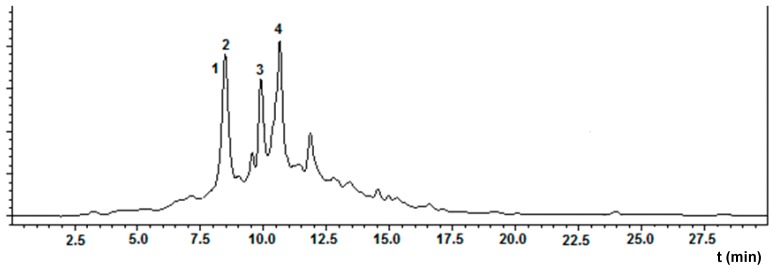
HPLC analysis of F.EtOAc.2 fraction of *P. friedrichsthalianum* fruit (C_18_-column, λ = 370 nm). Compounds: **1**: procyanidin B1; **2**: procyanidin B2; **3**: unidentified; and **4**: ellagic acid.

Procyanidin B1, procyanidin B2, and ellagic acid were found in the fraction F.EtOAc.2 (Figure 1) which exhibited the highest value of antioxidant activity (Table 1). Procyanidins B1 and B2 showed the protonated and deprotonated ions at *m*/*z* 579 and 577 in positive and negative modes, corresponding to the ions [M + H]^+^ and [M − H]^−^, respectively. Additionally, the ions at *m*/*z* 291 and 289 were detected, corresponding to the catechin monomer [14].

From the HPLC-ESI/MS (single quad) analysis (Figure 1) of the F.EtOAc.2 fraction, another phenolic compound (compound **3**) was detected. This compound showed the protonated and deprotonated ions at *m*/*z* 507 and 505 in positive and negative modes, respectively, thus suggesting a molecular weight of 506 u.

In order to elucidate the structure of these compounds, the antioxidant fraction F.EtOAc.2 was fractionated by using HyperSep™ C_18_-cartridges and subsequent preparative RRLC (rapid resolution liquid chromatography). This is a technique of liquid chromatography in which small particles are packed into short columns run with a small particle size and diameter, allowing a separation with higher resolution and sensitivity, and shorter retention times than the common HPLC [18].

**Figure 2:**
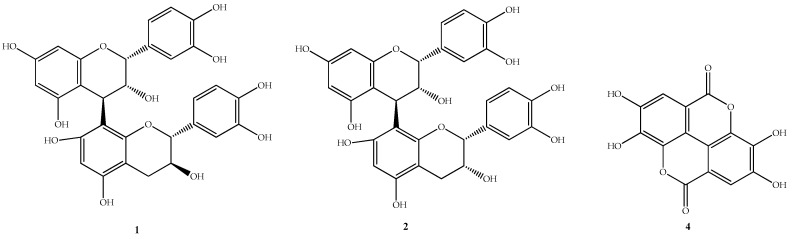
Structures corresponding to the identified compounds in the fractionation of the fraction F.EtOAc.2 of *P. friedrichsthalianum*: (**1**) procyanidin B1; (**2**) procyanidin B2; and (**4**) ellagic acid.

Compound **3** was then purified from subfraction F.EtOAc.2.3, which also exhibited the highest antioxidant value among all the compounds (Table 1). The ^1^H-NMR spectrum in DMSO of this compound showed signals of oxymethins from a sugar moiety at δ 3.42–3.80 ppm, with the anomeric proton at δ 5.12 ppm; the aromatic signals at δ 7.57 ppm (1H, s) and δ 8.07 ppm (1H, s); and three methoxyl signals at δ 3.40 (9H). However, with this information it was not possible to propose an unequivocal structure, only that is an ellagic acid-related derivative. The identity of compound **4** was confirmed by comparison with a standard sample (Figure 2).

The efficiency of ellagic acid and the procyanidins as antioxidant compounds has been attributed to the presence of the hydroxyl group in the ortho position which favors the ability to donate a hydrogen atom and support the unpaired electron. Ellagic acid is metabolized to urolithins, which have been reported as relevant bioactive compounds in the colon and may contribute to colon cancer chemo-preventive properties resulting from the consumption of foods containing this polyphenol [19]. In the case of procyanidins, several studies have reported their antioxidative properties, and B-type dimers were reported to inhibit LDL (low density lipoprotein) oxidation and were more potent than ascorbic acid and Trolox [20]. The other fractions with high antioxidant values, F.EtOAc.2.1 and F.EtOAc.2.4, were not studied because they exhibited a too-complex HPLC profile to get a good separation. F.EtOAc.2.5 was not further analyzed because it was obtained in a tiny amount.

## 3. Materials and Methods

### 3.1. Fruits

The fruits were acquired in local markets of Montería, Córdoba, Colombia, and selected according to their ripeness qualities. For this purpose, the pH of the pulp was determined by using a Jenway pH meter (model 370, Essex, England, UK), and total soluble solid content was determined by using an Atago refractometer (HRS-500, Tokyo, Japan). Titratable acidity was determined by the standard procedure [21] using 10 g of pulp, by triplicate, and the results expressed as percentage of citric acid. A voucher specimen was coded as COL 566221, identified, and deposited at the Instituto de Ciencias Naturales, Universidad Nacional de Colombia-Sede Bogotá.

### 3.2. Chemicals

The solvents used in LC-MS analyses were acetonitrile HPLC-MS grade (LabScan, Sowinskiego, Poland), formic acid (Sharlau, Barcelona, Spain) and water purified in a Milli-Q Waters Millipore (Milford, MA, USA). The other solvents (EMSURE^®^) were purchased from Merck (Darmstadt, Germany): acetonitrile, chloroform, methanol, acetone, ethyl ether, ethyl acetate, and *n*-butanol. The 63 standards used for tandem studies in UHPLC-ESI/QqQ were acquired in different trademarks, such as Sigma-Aldrich Chemical Co. (St. Louis, MO, USA), EXTRASYNTHESE (Genay, France), and Phytolab (Vestenbergsgreuth, Germany).

### 3.3. Extraction

Whole fruits (2416 g) were cut into pieces and freeze-dried to obtain 545 g of dried fruit. Lyophilized whole fruits (388 g) were extracted with three volumes (500 mL) of acetone:water (7:3), and then, the extracts were combined and concentrated to one liter volume. This aqueous extract was submitted to successively partitions with ethyl ether, ethyl acetate, and *n*-butanol, according to the procedure reported by Isaza et al. [13]. For extraction, 300 mL of each solvent was used in triplicate. In all fractions, the solvent was distilled off under reduced pressure and subsequent freeze-drying, obtaining 6.9 g of ethyl ether fraction (F.Ether), 5.9 g of ethyl acetate fraction (F.EtOAc), 29.1 g of butanol fraction (F.BuOH), and 200.8 g of aqueous fraction (F.aqueous). Each of these fractions was analyzed by HPLC-MS.

### 3.4. Determination of Total Phenolic Content by Folin-Ciocalteou Method

Total phenolic content was determined by the spectrophotometric method Folin-Ciocalteou, using gallic acid (Merck, Damstadt, Germany) as standard [22]. A solution was prepared by a 1:10 dilution of commercial reagent (Merck, Damstadt, Germany) in distilled water; the reagent was protected from light and placed under refrigeration until use. For each sample, 100 µL of sample or standard and 0.5 mL of Folin solution were placed in a 1 mL cuvette and after four minutes the reaction was stopped by adding 400 µL of Na_2_CO_3_ saturated solution. Then, the samples were left at 18 °C in a dark place by 2 h. Finally the absorbance was measured in a spectrophotometer UV-Vis Jenway 7305 (Burlington, VT, USA) at λ 760 nm. The assays were performed in triplicate, and total phenol content was expressed in mg of gallic acid equivalents (GAE)/100 g of fruit.

### 3.5. Determination of Antioxidant Activity by ABTS Assay

This method is based on the ability of the compounds to scavenge ABTS^•+^ radical cation. The assay was performed according to the method developed by Re, et al. [23]. The ABTS^•+^ cation radical is produced by the reaction between ABTS (7 mM) and potassium persulfate (2.45 mM) in 10 mL of distilled water. Then, this solution was diluted in methanol to obtain the working solution (0.18 mM), with an absorbance of ca. 0.7 at λ 734 nm. Trolox standard solutions (Fluka Chemie GmbH, Steinheim, Switzerland) were prepared in ethanol at concentrations of 0.5, 1.0, 1.5 and 2 mM. For the determination of antioxidant activity, 1 mL of the working solution was added 10 µL of each sample and the absorbance was measured 6 min after, in a spectrophotometer UV-Vis Jenway 7305 (Burlington, VT, USA) at λ 734 nm. The data were interpolated in the curve and expressed as TEAC (Trolox-equivalent Antioxidant Capacity). The TEAC value is defined as mmol Trolox equivalents per kg of fresh fruit. The ascorbic acid (Merck, Darmstadt, Germany) was used as positive reference compound and the assays were performed in triplicate.

### 3.6. Targeted Metabolomic Analysis of Phenolic Compounds

A targeted analysis for the detection, identification, and quantitation of phenolic compounds was performed by UPLC-ESI/QqQ following the methodology previously described [16,17]. An UPLC system coupled to an Acquity PDA photodiode array detector and an Acquity TQD tandem quadrupole mass spectrometer (UPLC-PAD-ESI/TQ/MS) (Waters, Milford, MA, USA) was used. Chromatographic separations were performed on a Waters UPLC BEH C_18_-column (2.1 × 100 mm, 1.7 μm i.d.), operating at a temperature of 40 °C. The solvent system was a mixture of water/formic acid (99.9:0.1, *v*/*v*, solvent A) and acetonitrile/formic acid (99.9:0.1, *v*/*v*, solvent B) at the flow rate of 0.5 mL/min. A linear gradient from: 0.1% B at 1.5 min, 0.1% to 16.3% B during 1.5–11.17 min, 16.3% to 18.4% during 11.17–11.5 min, 18.4% to 99.9% during 11.5–14.1 min., and 99.9% to 0.1% during 14.1–15.6 min, was used. The injection volume was 2 µL. Detection parameters of MS was: capillary voltage, 3 kV; source temperature, 130 °C; desolvation temperature, 400 °C; desolvation gas flow (N_2_), 750 L/h; cone gas flow (N_2_) 60 L/h; ionization source, negative mode (ESI^−^).

The detection was performed in multiple reaction monitoring mode (MRM), and the parameters were adjusted using standard 63 mostly phenolic-type or degradation products [15]. To do so, in previous works for all the compounds, the MS/MS parameters (cone voltage and collision energy) of each analyte were initially optimized by direct infusion experiments, and for each of them, the most sensitive transition (precursor and product ions) was selected for quantification purposes using the MRM mode [16,17]. Specifically, the MRM transitions used to detect flavan-3-ols were: (+)-catechin and (−)-epicatechin (289 > 245), procyanidins B1 and B2 (577 > 289), procyanidin A2 (575 > 449). MRM transitions used to detect phenolic compounds of low molecular weight were: tyrosol (137 > 106), phloroglucinol (125 > 83), pyrogallol (125 > 79), catechol/pyrocatechol (109 > 81), 4-methylcatechol (123 > 108), 4-ethylcatechol (137 > 122), resveratrol (227>185). MRM transitions used to detect phenylpropanoic acid derivatives were: 3-(3,4-dihydroxyphenyl) propanoic acid (181 > 137), 3-(4-hydroxyphenyl propanoic acid) (165 > 121), 3-(3-hydroxyphenyl propanoic acid) (165 > 121), 3-(3,4-dimethoxyphenyl propanoic acid) (209 > 150) phenylpropanoic acid (149 > 105). MRM transitions used to detect phenylacetic acid derivatives were: phenylacetic acid (135 > 91), 3,4-dimethoxy acid (195 > 136), 4-methoxyphenylacetic acid (165 > 106), 3-hydroxyphenylacetic acid (151 > 107), 4-hydroxy-3-methoxyphenylacetic acid (181 > 137), 4-hydroxyphenylacetic acid (151 > 107), 3,4-dihydroxyphenylacetic acid (167 > 123). MRM transitions used to detect benzoic acid derivatives were: gallic acid (169 > 125), salicylic acid (137 > 93), syringic acid (197 > 182), 3,5-dihydroxybenzoic acid (153 > 109), protocatechuic acid (153 > 109), 4-hydroxybenzoic acid (137 > 93), 3-hydroxybenzoic acid (137 > 93), vanillic acid (167 > 152), 3,4-dimethoxybenzoic acid (181 > 107), benzoic acid (121 > 77), 4-methoxybenzoic acid (151 > 107), 3,4,5-trimethoxybenzoic acid (211 > 167), 3-methoxybenzoic acid (151 > 107), caffeic acid (179 > 135), 3,4-dihydroxymandelic acid (183 > 137), 4-hydroxymandelic acid (167 > 123), 4-hydroxy-3-methoxymandelic acid (197 > 137), 3-hydroxy-4-methoxymandelic acid (197 > 137), 3-hydroxymandelic acid (167 > 121), mandelic acid (151 > 107), 3-*O-*methylgallic acid (183 > 168), 4-*O*-methylgalic acid (183 > 168), 4-hydroxyhippuric acid (194 > 100), *p*-coumaric acid (163 > 119), *m*-coumaric acid (163 > 119), isoferulic acid (193 > 134), ferulic acid (193 > 134), ellagic acid (301 > 145). MRM transitions used to detect valerolactones derivatives are α-valerolactone (101 > 55) di-hydroxyphenyl valerolactone (207 > 163), hydroxyphenyl valerolactone (191 > 147). MRM transitions used to detect valeric acid derivatives were: di-hydroxyphenyl-4-hydroxyvaleric acid (225 > 163), hydroxyphenyl-4-hydroxyvaleric acid (209 > 147), phenyl-4-hydroxyvaleric acid. MRM transitions used to detect other types of aromatic compounds were: phthalic acid (165 > 121), dihydroxyphenyl-2-propanol (291 > 247), 3,4,5-trimethoxycinnamic acid (237 > 103), *trans*-cinnamic acid (147 > 103), hippuric acid (178 > 134). For quantitation, the data were collected in multiple reaction mode (MRM), following the transition of parent ions and specific for each product compound, and the curves were constructed using linear concentrations in µg/mL. The data analysis was performed using the MassLynx (Waters, Milford, MA, USA) [16]. All injections were performed in duplicate.

### 3.7. LC-MS Analyses

The fractions obtained by partition of *P. friedrichstalianum* polar extract were analyzed in a liquid chromatograph LC-10AD Shimadzu (Kyoto, Japan) coupled to mass spectrometer Shimadzu LCMS-2010 EV (Kyoto, Japan). The injection of samples was performed in a loop injector Rheodyne 5 µL. A C_18_-column Shimadzu-5 µm (50 × 4.6 mm i.d.) was used. The mobile phase was acetonitrile–formic acid 0.1% (solvent A) and formic acid–H_2_O 0.1% (solvent B). The samples were eluted with a linear gradient which started with 5% to 60% of solvent A in 27 min; the solvent flow was set at 0.4 mL/min. The eluted compounds were monitored at two wavelengths λ 280 and 370 nm. The mass spectrometer was operated in positive and negative electrospray ionization (ESI) mode, 1.5 kV, CDL 300 °C, block 240 °C, gas flow (N_2_) to 1.5 L/min, CDL voltage 150 kV, Q array RF voltage 150 V, and detector voltage 1.5 kV. The positive and negative ions were detected between *m*/*z* 50 and 1000 u.

### 3.8. Purification of Compound ***3***

The fraction obtained by partition with ethyl acetate (F.EtOAc, 5.9 g), was fractionated by size exclusion chromatography, using Toyopearl HW-40S (Tosoh, PA, USA, 30 µm particle size, 11.5 × 3 cm) as stationary phase, according to the procedure published by Isaza et al. [13]. Four fractions were obtained: F.EtOAc.1 was eluted with MeOH/water (2:3, *v*/*v*), F.EtOAc.2 with MeOH/water (7:3, *v*/*v*), F.EtOAc.3 with acetone/MeOH/water (2:5:3, *v*/*v*/*v*), and finally F.EtOAc.4 with acetone/water (7:3, *v*/*v*). For each fraction, 100 mL of solvent mixture were used and distilled off under reduced pressure and subsequent freeze-drying. All fractions were analyzed by HPLC-MS as it was explained in Section 3.7.

Fractionation of F.EtOAc.2 was performed by column chromatography (CC) under vacuum using C_18_-cartridges HyperSep™ (Thermo Scientific, Waltham, MA, USA, 40–60 µm particle size, 1 g bed weight). A discontinuous gradient of polarity was used (water, MeOH/water (1:4, *v*/*v*), MeOH/water (2:3, *v*/*v*), MeOH/water (3:2, *v*/*v*), MeOH/water (4:1, *v*/*v*), MeOH, acetone), obtaining seven subfractions (F.EtOAc.2.1–F.EtOAc.2.7). All fractions were analyzed by HPLC-MS and their antioxidant activity was determined by the ABTS assay (see Section 3.5) to continue the fractionation.

The F.EtOAc.2 fraction (50 mg) was further fractionated by preparative HPLC on Agilent 1260 HPLC (Agilent Technologies, Santa Clara, CA, USA). An Agilent column, Poroshell 120, SB-C_18_ (2.7 µm, 4.6 i.d × 150 mm) was used. Rheodyne loop injector of 20 µL was used. The mobile phase was acetonitrile–formic acid 0.1% (solvent A) and formic acid–H_2_O 0.1% (solvent B), and the samples were eluted in isocratic mode with solvent A 25% during 25 min. The flow was set at 0.5 mL/min and compounds were monitored at λ 280 and 370 nm. Ten subfractions were obtained (F.EtOAc.2.3.1–F.EtOAc.2.3.10). From F.EtOAc.2.3.5, the compound **3** was purified (24.8 mg).

The pure compound was analyzed by HPLC-MS and ^1^H-NMR. The NMR spectra were recorded on a Bruker Avance 400 (400 MHz), using DMSO-d_6_ with deuteration degree 99.9% as solvent, and signals referenced to the residual non-deuterated solvent signal at δ_H_ 2.50 ppm. The results were processed and analyzed with MestReNova 8.1 software (Mestrelab Research SL, San Diego, CA, USA).

### 3.9. Statistic Analysis

The results obtained in the quantitation of phenolic compounds, the determination of total phenolic content by Folin-Ciocalteou and antioxidant activity, were subjected to analysis of variance and Tukey test (*p* < 0.05) to determine significant difference; the averages and standard deviation were calculated taking into account the ISO 3534-1 [24].

## 4. Conclusions

The identity of some polyphenols in *P. friedrichsthalianum* was confirmed by a targeted metabolomic approach. Ellagic acid, procyanidin B1 and procyanidin B2 are reported here as some of the compounds responsible for the antioxidant activity of this fruit under ABTS assay. This is the first time that (+)-catechin, procyanidin B1, procyanidin B2, and (−)-epicatechin have been reported as constituents of sour guava. These phenolic compounds are well recognized to exhibit a wide range of bioactivities including antioxidant, anticancer, and anti-inflammatory activities, among others. The results showed a potential of *P. friedrichstalianum* as a source of antioxidant compounds for the development of functional foods, because the total phenolic content in the ethyl acetate fraction is comparable to that of common guava (*Psidium guajava*) fruit and higher than those reported for other tropical fruits [25] and other non-tropical fruits such as apple, peach and tomato [26].

## Figures and Tables

**Table 1:** Total phenolic content and antioxidant activity of *P. friedrichsthalianum* fractions.

Fraction	Amount Isolated (g)	Concentration of Total Phenolics (mg GAE/100 g Fruit ± SD) *	Antioxidant Activity (mM Trolox Equivalent/kg Fruit ± SD) *
F.Ether	6.9	25.94 ± 0.05 ^a^	0.16 ± 0.09 ^a^
F.EtOAc	5.9	138.08 ± 0.07 ^b^	0.85 ± 0.05 ^b^
F.BuOH	29.1	71.95 ± 0.05 ^c^	0.14 ± 0.01 ^a^
F.aqueous	200.8	92.32 ± 0.02 ^d^	0.02 ± 0.00 ^c^
F.EtOAc.1	3.4	-	2.08 ± 0.11 ^d^
F.EtOAc.2	1.0	-	10.99 ± 1.09 ^e^
F.EtOAc.3	0.8	-	0.66 ± 0.01 ^f^
F.EtOAc.4	0.6	-	0.40 ± 0.21 ^g^
F.EtOAc.2.1	0.186	-	3.19 ± 0.13 ^h^
F.EtOAc.2.2	0.199	-	0.22 ± 0.01 ^i^
F.EtOAc.2.3	0.406	-	4.76 ± 0.22 ^j^
F.EtOAc.2.4	0.226	-	3.09 ± 0.15 ^h^
F.EtOAc.2.5	0.044	-	3.14 ± 0.04 ^h^
F.EtOAc.2.6	0.020	-	2.76 ± 0.35 ^h^
F.EtOAc.2.7	0.003	-	2.84 ± 0.46 ^h^

- Not determined. * Values are expressed as mean ± SD (*n* = 3); GAE: gallic acid equivalents. ^a–j^ Values in the same column/group followed by different letters are significantly different by ANOVA test (*p* < 0.05). F.Ether (ether fraction), F.EtOAc (ethyl acetate fraction), F.BuOH (butanol fraction), and F.aqueous (aqueous fraction).

**Table 2:** Identification and quantitation of phenolic compounds in F.AcOEt fraction of *P. friedrichsthalianum* fruit by UHPLC-ESI/QqQ (negative mode).

Compound	Amount (mg/g Fraction)	Retention Time (min)	Molecular Mass (u)	MRM Transition	Calibration Range (μg/mL) *	LOD (μg/mL) *	LOQ (μg/mL) *
*Benzoic acids and derivatives* (*C*_6_*–C*_1_)							
Benzoic acid	0.276 ± 0.029	8.49	122.12	121 > 77	12.50–0.39	0.090	0.200
4-Hydroxybenzoic acid	0.002 ± 0.000	4.43	138.12	137 > 93	3.13–0.06	0.012	0.025
Protocatechuic acid	0.081 ± 0.006	3.34	154.12	153 > 109	3.13–0.02	0.006	0.012
Vanillic acid	0.204 ± 0.005	5.43	166.14	165 > 121	25.12–0.39	0.050	0.098
Phthalic acid	0.023 ± 0.002	5.34	166.14	165 > 121	3.13–0.02	0.012	0.025
Gallic acid	0.693 ± 0.010	1.73	170.12	169 > 125	1.56–0.05	0.012	0.025
3-*O*-Methylgallic acid	0.007 ± 0.001	4.22	184.15	183 > 168	1.56–0.01	0.007	0.016
Syringic acid	0.040 ± 0.010	5.95	198.17	197 > 182	1.56–0.10	0.012	0.030
*Phenylacetic acids* (*C*_6_*–C*_2_)							
Phenylacetic acid	1.087 ± 0.088	8.77	136.15	135 > 91	12.50–0.78	0.390	0.780
3,4-Dihydroxyphenylacetic acid	0.001 ± 0.000	4.16	168.15	167 > 123	0.39–0.01	0.003	0.007
4-Hydroxymandelic acid	0.092 ± 0.001	1.42	168.15	167 > 123	6.25–0.20	0.098	0.196
*Phenylpropanoic acids and derivatives* (*C*_6_*–C*_3_)							
Caffeic acid	0.020 ± 0.005	5.46	180.16	179 > 135	3.13–0.05	0.024	0.049
Isoferulic acid	0.020 ± 0.007	8.40	194.18	193 > 134	0.78–0.05	0.024	0.049
Ferulic acid	0.021 ± 0.002	7.81	194.18	193 > 134	3.13–0.02	0.006	0.012
*Flavan-3-ols* (*C*_6_*–C*_3_*–C*_6_)							
(+)-Catechin	17.952 ± 1.404	5.22	290.26	289 > 245	6.25–0.05	0.012	0.024
(−)-Epicatechin	9.525 ± 1.581	6.36	290.27	289 > 245	3.13–0.05	0.018	0.049
Procyanidin A2	0.049 ± 0.002	9.02	576.51	575 > 449	3.13–0.05	0.006	0.024
Procyanidin B1	14.149 ± 1.454	4.91	578.52	577 > 289	25.00–0.20	0.050	0.1000
Procyanidin B2	10.820 ± 1.122	5.94	578.52	577 > 289	25.00–0.39	0.030	0.060
*Others* (*Phenols*, *lactones*)							
α-Valerolactone	0.024 ± 0.014	3.45	100.12	101 > 55	1.56–0.05	0.024	0.098
Pyrogallol	0.059 ± 0.008	1.69	126.11	125 > 79	1.56–0.05	0.024	0.049
Ellagic acid	4.755 ± 0.105	14.61	302.20	301 > 145	50.00–1.56	0.268	0.521

* Gall, E. [15]; The MRM transitions were those already optimized and published in previous works [16,17]. LOD = Limit of Detection, LOQ = Limit of Quantitation.
